# Saliva-based microfluidic point-of-care diagnostic

**DOI:** 10.7150/thno.78872

**Published:** 2023-01-31

**Authors:** Trey W. Pittman, Daniel Balazs Decsi, Chamindie Punyadeera, Charles S. Henry

**Affiliations:** 1 Department of Chemistry, Colorado State University, Fort Collins, Colorado 80523, USA.; 2 Centre for Biomedical Technologies, School of Biomedical Sciences, Faculty of Health, QUT.; 3 Griffith Institute for Drug Discover, Griffith University, Nathan, Australia.; 4 Menzies Health Institute, Griffith University, Gold Coast, Australia.; 5 Translational Research Institute, Woolloongabba, Australia.; 6 Metallurgy and Materials Science Research Institute, Chulalongkorn University, Soi Chula 12, Phayathai Rd., Pathumwan, Bangkok 10330, Thailand.

**Keywords:** Saliva, Diagnostics, point-of-care, lab-on-chip, microfluidic.

## Abstract

There has been a long-standing interest in point-of-care (POC) diagnostics as a tool to improve patient care because it can provide rapid, actionable results near the patient. Some of the successful examples of POC testing include lateral flow assays, urine dipsticks, and glucometers. Unfortunately, POC analysis is somewhat limited by the ability to manufacture simple devices to selectively measure disease specific biomarkers and the need for invasive biological sampling. Next generation POCs are being developed that make use of microfluidic devices to detect biomarkers in biological fluids in a non-invasive manner, addressing the above-mentioned limitations. Microfluidic devices are desirable because they can provide the ability to perform additional sample processing steps not available in existing commercial diagnostics. As a result, they can provide more sensitive and selective analysis. While most POC methods make use of blood or urine as a sample matrix, there has been a growing push to use saliva as a diagnostic medium. Saliva represents an ideal non-invasive biofluid for detecting biomarkers because it is readily available in large quantities and analyte levels reflect those in blood. However, using saliva in microfluidic devices for POC diagnostics is a relatively new and an emerging field. The overarching aim of this review is to provide an update on recent literature focused on the use of saliva as a biological sample matrix in microfluidic devices. We will first cover the characteristics of saliva as a sample medium and then review microfluidic devices that are developed for the analysis of salivary biomarkers.

## Introduction

The outbreak of SARS-CoV-2-induced COVID-19 has demonstrated how rapidly a new pathogen can spread from a single city outbreak to global scale pandemic, that devastated the healthcare sector 1-4. While acute coronavirus disease is predominantly detected using nasal swab samples coupled with reverse transcription-polymerase chain reaction (RT-PCR) for the detection of viral genetic material. Evidence suggests that SARS-CoV-2 infection is detectable within the oral cavity as the oral axis of virus pathogenesis and transmission has been further explored 5-7. There is growing evidence of literature to suggest that salivary testing can complement the current nasal testing methods. Huang et al (2021), identified that nasal swab false negative COVID-19 cases were detectable using saliva testing 5. Further, prestigious studies have explored the viability of using saliva as a matrix for COVID-19 disease analysis and have concluded that saliva is consistently a better alternative to nasopharyngeal and nasal swabs 8-10.

A key weakness in the healthcare sector that was discovered during the pandemic was the inability to perform high through put testing and the lack of methods to perform remote and field testing to stop the spread of the virus. Though these issues were mediated with temporary expansion of staff and testing facilities, in the event of another disease outbreak to the scale of SARS-CoV-2, a shortfall of field-testing capacity is likely to become a problem. The pandemic further revealed that the capacity of a nation to detect the spread of an infection throughout a community and identify its source was paramount to successfully controlling the spread before it becomes unmanageable 11. The deployment of rapid antigen testing (RAT) kits has served as a turning point during the height of the pandemic by allowing testing to be carried out within a home-setting, minimizing the threat of spreading the pathogen. This revolutionization of healthcare by POC technology has been demonstrated in the past through the urine-based test to measure glucose for diabetes management. Again, two decades later, the commercialization of an at-home pregnancy test, that has become the most widely used POC 12.

An early diagnosis of a disease is vital to enable early medical intervention to efficiently manage a patient and ensure the best possible outcome for a person. Currently, central laboratory-based serological testing remains the most widely used method of analytical testing 13. Due to the limitations and pressure on the current healthcare systems, there is an urgent need for non-invasive testing. Saliva-based POC testing is one of the best options to increase accessibility, reduce expenses through early diagnosis of diseases and enable early treatment. The transition to salivary diagnostics is attractive because while upholding current testing standards, sample collection is non-invasive and risk free when compared to blood-based methods, leading to an increase in patient compliance for testing 14-16. As seen during the current pandemic, it is clear that testing is required to be done in a remote or field setting to minimize the widespread of infection. Saliva based POC devices meet this unmet clinical need, while enhancing bedside patient monitoring. Saliva is also a more stable and a less complex matrix compared to blood and as such, is ideal for field testing. Saliva has been championed as the diagnostic fluid of the future over blood and urine and microfluidic technology offers deployability, while remaining cost-effective and upholding testing integrity.

The overarching objective of this research article is to comprehensively review the current literature on saliva based microfluidic devices. Saliva is gaining traction as a biological fluid that is being explored to replace the conventional serological tests. For saliva to replace blood-based testing for diagnosing and monitoring of systemic diseases, it must first demonstrate that the underlying disease pathogenies can be accurately captured using saliva. Secondly, it must be able to demonstrate clinical utility and adaptability of saliva testing for disease diagnosis, prognosis and surveillance. Thirdly, there is paucity of data relating to saliva based POC testing as true standalone, portable devices. We envision that integration of saliva sampling with microfluidic POC technologies will undoubtedly address this current unmet need. To the best of our knowledge, the application of saliva as a biological fluid with POC technologies lacks in depth literature review describing the complexities of combining these two advancing fields 13, 17. Our review article will address this gap in knowledge by exploring the potential of saliva as a diagnostic body fluid, the complexities of using saliva on microfluidic devices and the advancement of microfluidic POC devices from a biological and engineering perspectives. Salivary diagnostics can facilitate decentralized, miniaturized integrated systems that can be coupled with mobile testing interfaces to increase testing capacity and enable the monitoring of people living in rural and remote communities as well as in hospital settings.

## Saliva as a diagnostic fluid for the detection of oral and systemic diseases

Saliva is a complex, viscoelastic fluid that contains a large number of biomolecules, which includes enzymes (e.g., α-amylase), hormones, antibodies, and antimicrobial components 18, 19. Biochemical studies have revealed that saliva contains both organic (glycoproteins, immunoglobulin alpha, enzymes, lactoferrin, amylase, mucins, lysozyme, histatins, cathelicidins, defensins, glycoproteins, lipoproteins, statherin, and matrix metalloproteases) and inorganic molecules (sodium, potassium, calcium, magnesium, chloride, and phosphate) 19, 20. The salivary proteome consists of approximately 2000 unique proteins and peptides that can be quantified to diagnose a multitude of pathologies 18, 21-24. About 27% of these proteins are also found in blood, opening up opportunities to use saliva as a preferred diagnostic fluid 24-26.

There is now ample evidence linking oral health to systemic diseases 27. Salivary biomarkers have been used to diagnose head and neck cancer, breast cancer, heart failure, periodontal disease, salivary gland diseases, drugs of abuse and COVID-19 28-40. These applications are still in a research phase. Saliva is also considered as a step-child to mainstream blood-based tests, in terms of clinical utility because of the low levels of biomarkers in saliva (often 1000-fold lower) when contrasted to blood as a counterpart 39. The advancement of modern analytical techniques enables researchers to quantify biomolecules in saliva at very low analyte levels (pg/mL levels) 39, 41. This is a major advancement in the field and facilitates extremely sensitive quantitative measures. In recent years, salivary diagnostics have extended to many clinical applications, including road-side testing for illicit drugs, analyzing salivary hormones and protein levels to determine an athlete's performance status, and many clinical applications still in research setting for diagnosing cancers and viral infections 32, 38, 42, 43.

Saliva, as a diagnostic fluid is attracting increasing attention due to its non-invasive nature and the ease of sample collection, leading to greater patient compliance compared to blood-based methods 39. As saliva collection is minimally invasive, studies have recorded greater compliance using saliva when compared to blood samples 18. Saliva collection is ideal for elderly and young patients, as it imposes minimal discomfort to the donor, while remaining cost-effective, and as such patient compliance is notably increased 18. The collection of biological specimens in a non-invasive and patient-centric method is especially important when measuring stress parameters, as 'needle anxiety' intrinsically alters stress hormones and thus produces a false positive increase in cortisol levels 44. Furthermore, the collection of saliva imposes no health risk for the patient or the specimen collector as sample collection is non-invasive 45. Saliva further enables the ethical collection of a diagnostic specimen in challenging circumstances, such as cultural dissuasion, physical limitation, mental obstacles and conditions where traditional blood collection could impose unnecessary risks by breaking the skin barrier (hemophilia and immunocompromised) 18, 45. Saliva also offers greater accessibility to diagnostic testing as it requires less preprocessing when compared to blood, which requires fraction separation methods for a multitude of assays. Saliva can become an ideal diagnostic medium for disease detection when compared to blood as it enables for the long-term storage of unprocessed specimens indefinitely, if stored below 0°C for transport and at -80°C for long term storage to eliminate enzyme degradation, protein denaturization and the formation of artifacts 46, 47. While, standard EDTA and SST tubes can only maintain sample integrity for limited time (<2 hours at room temperature and up to 12 hours if refrigerated), saliva can be collected in DNA/RNA shield buffers for temporary storage and transport at room temperature, as demonstrated by Dahlén *et al*., (1993) using VMGa III media 48, 49. Thus, salivary testing is more accessible due to reduced requirements for equipment and is ideal for field testing.

With the overwhelming advantages of utilising saliva as a diagnostic medium for medical testing, regulated procedures are needed to be established prior to its uptake in a diagnostic setting 50. To better regulate and establish the consistent performance of saliva, collection methods must be standardized to ensure consistent collection of specimens, aided by collection devices 50. Once the application of saliva as a diagnostic medium is standardised, this will have an impact in developing multitude of diagnostic tools. The application of salivary diagnostics can complement current medical procedures with less-invasive, more rapid, and accessible protocol implementations. Furthermore, because of its non-invasive nature of sampling, saliva sampling could become an important diagnostic medium to advance medical testing both in populations from suburban and rural communities through the development of highly specified, rapid, field on chip devices (FOC).

## The production of saliva

On average, a healthy adult produces between 500 mL and 1500 mL of saliva every day 39, 51, 52. The specific rate of salivary flow is dependent on several physiological and pathological conditions 53. Saliva is made by three major and minor salivary glands, which are located in the underlying tissues of the oral cavity. Acini cells are the basic units of salivary glands and are involved in saliva production. The second major cell type of significance for saliva production is ductal cells 54. Saliva production is controlled by the autonomic nervous system, which controls both the volume and type of saliva being secreted 54, 55. The secretion of saliva by each gland is controlled by sympathetic and parasympathetic nerves. During the day, the parasympathetic nerve supply is most active and creates waterier, or serous saliva; predominantly produced by the parotid gland and to a lesser extent by submandibular gland 27. The parasympathetic system elicits the increased rate of saliva flow by releasing acetylcholine, which stimulates the glands to make more saliva 54. If the glands become diseased, damaged, or affected by drugs, saliva production is subsequently affected, often causing reduced production. During fear, stress, or anger stimulation, sympathetic nerves are more innervated causing a cascade that reduces the blood supply to our digestive system causing the sublingual and submandibular glands to produce more viscous 'mucous saliva' in the physiological state. Multi-constituent mucinous and serous whole saliva is a complex mixture of nucleic acids, proteins, enzymes, peptides, immune cells, hormones, electrolytes, molecules from blood, salts, and water 50, 56. Consequently, each gland produces a specific type of saliva, which is dependent on the rheological properties of the producing salivary gland 39. The parotid gland produces saliva that is rich in alpha-amylase, but low in other proteins with a low viscosity (1-3 mPa), resembling water 57. In contrast, the submandibular gland produces a mixture of serious and mucous type saliva that is more viscous, facilitating early digestive processes 57.

## Transport of biomolecules between blood and saliva

The salivary glands are encircled by dense beds of capillaries, and many blood factors enter easily through the capillary walls, immersing the saliva glands. Consequently, there is continuous biomolecular transport across salivary acinar cells and blood endothelium cells. There is growing evidence to support that oral health is an indicator of systemic health, posing saliva as a prospective biofluid for discerning the general health of an individual 21, 23, 58. For saliva to be a suitable analytical matrix for systemic disease diagnosis, it must have a continuous flow of biomolecules across the blood endothelium cells to salivary gland acinar cells at reasonable flow rates. As an example, Foo *et al.,* (2012) quantified cardiac specific biomarker N-terminal pro b-type natriuretic peptide (NT-proBNP) in saliva collected from age-matched healthy controls and patients with heart failure (HF). The median NT-proBNP levels in the saliva samples from healthy controls and HF patients were <16 pg/mL and 76.8 pg/mL, respectively 41. The salivary NT-proBNP immunoassay showed a clinical sensitivity of 82.2% and specificity of 100%, positive predictive value of 100% and negative predictive value of 83.3%, with an overall diagnostic accuracy of 90.6% 41.

**Figure 2:**
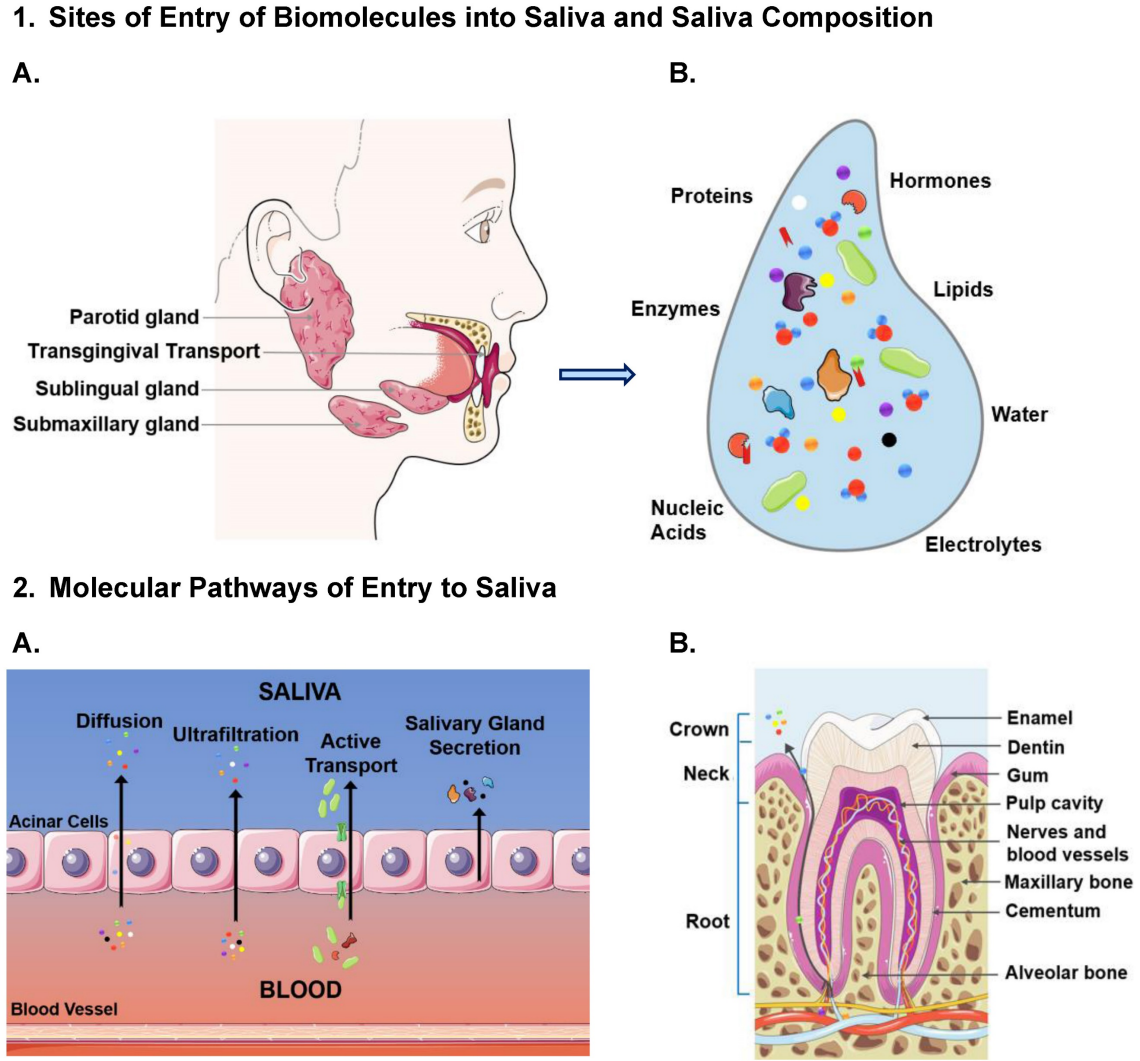
** A schematic representation of biomolecular transport between salivary glands and blood endothelium cells. (1A)** Displays the dominant salivary production sites and locations where circulatory molecules enter saliva. **(1B)** The composition of saliva is then visualized to represent its complex molecular makeup with various biomolecules. 28. **(2)** Consists of two images that illustrate the modalities of entry for molecules into saliva: **(A)** The passive diffusion of small and neutral biomolecules through acinar cells. The ultrafiltration of molecules below 190kDa that passage between the gap junctions between acinar cells. The active transport of large molecules that exceed 190kDa through cell mediated, selective, active mechanisms 33, 39. B represents the entry of molecules between the transgingival junction via diffusion. The figure was created using Servier Medical Art templates, licensed under a Creative Commons Attribution 3.0 Unported License; https://smart.servier.com.

Depending on the type of the disease, biomolecule transport across salivary gland acinar cells and endothelium cells underlying blood vessels may be impacted. As such, there will be alterations in the abundance of disease specific biomolecules. The mechanism of entry of these constituents from the blood into the saliva is thought to be facilitated by transcellular, passive intracellular diffusion and active transport, or paracellular routes by extracellular ultrafiltration within the salivary glands or through gingival crevices (as illustrated in Figure 2) 28. The transport mechanism of specific molecules into saliva is dependent on their size and charge 33. As an example, electrically charged steroids such as dehydroepiandrosterone sulfate (DHEA-S) are not able to diffuse through the neutral lipid membranes of the salivary cells. Thus far, the mode of entry for DHEA-S into saliva is not clear. The most common routes of molecule transport into saliva from blood involve passive diffusion. For instance, neutral steroids diffuse readily through the lipoprotein cell membranes of the secretory cells in the saliva glands and into saliva. Studies have shown that the speed of entry is rapid, and that stimulation of saliva flow does not affect the concentrations of these neutral steroids entering salivary circulation. This process is especially relevant for the diffusion of steroid hormones, as their fatty acid molecular structure render them non-polar 39. Serum proteins like albumin or immunoglobins are too large to pass through the membranes of the salivary cells and are believed to be transported via active transport by transmembrane protein ligands. This process is selective to molecules that cannot diffuse passively through the membrane, such as B-lymphocyte secreted immunoglobulins (IgA and IgG), and molecules that exceed 60 kDa in size 59-61. Another mechanism by which ions and unconjugated steroid molecules are transported to saliva from the blood vessels involves ultrafiltration, where low molecular weight biomolecules, between 100-200 kDa, are transported between the gap junctions of acinus and ductal cells 17, 39, 60, 62. The quantity of transfer of said molecules is highly dependent on the rate of the flow of the saliva, resulting in a reduction in abundance (100-3000-fold reduction) of molecules compared to serum levels 39, 62.

## Saliva collection devices

The most common saliva collection method from participants includes passive drool saliva or unstimulated whole mouth saliva collection. This is conducted by asking participants to sit in a comfortable position, tilt their heads down and to pool saliva in the mouth prior to collection 23, 39, 63, 64. Moreover, whole mouth saliva can be targeted by the passage of or facilitating liquid (i.e., 10 mL of saline solution or distilled water) through an individual's mouth. This method of saliva is usually used when interested in diagnosing oropharyngeal cancers associated with human papillomavirus infection or any other virus residing in the tonsillar region 30, 65. When a patient's salivary glands are affected by either an underlying disease or treatment, alternative methods are required. To address this issue, researchers have used alternative methods to collect saliva by stimulating salivary glands to produce stimulated saliva. For the collection of mechanically and acid stimulated saliva, the mechanical induction of salivation is to be invigorated by chewing on paraffin wax or rubber bands or by applying a flavour (such as a citric acid 0.1-0.2 mol/L) or odour stimulant 39. In addition, saliva secretion can be selectively targeted by canulating the desired salivary gland ducts or using collection devices specifically designed for the specified saliva collection 66. As an example, saliva from parotid glands can be collected by using a modern version of the Carlson-Crittenden device.

## Commercially available saliva collection devices

**Figure 3:**
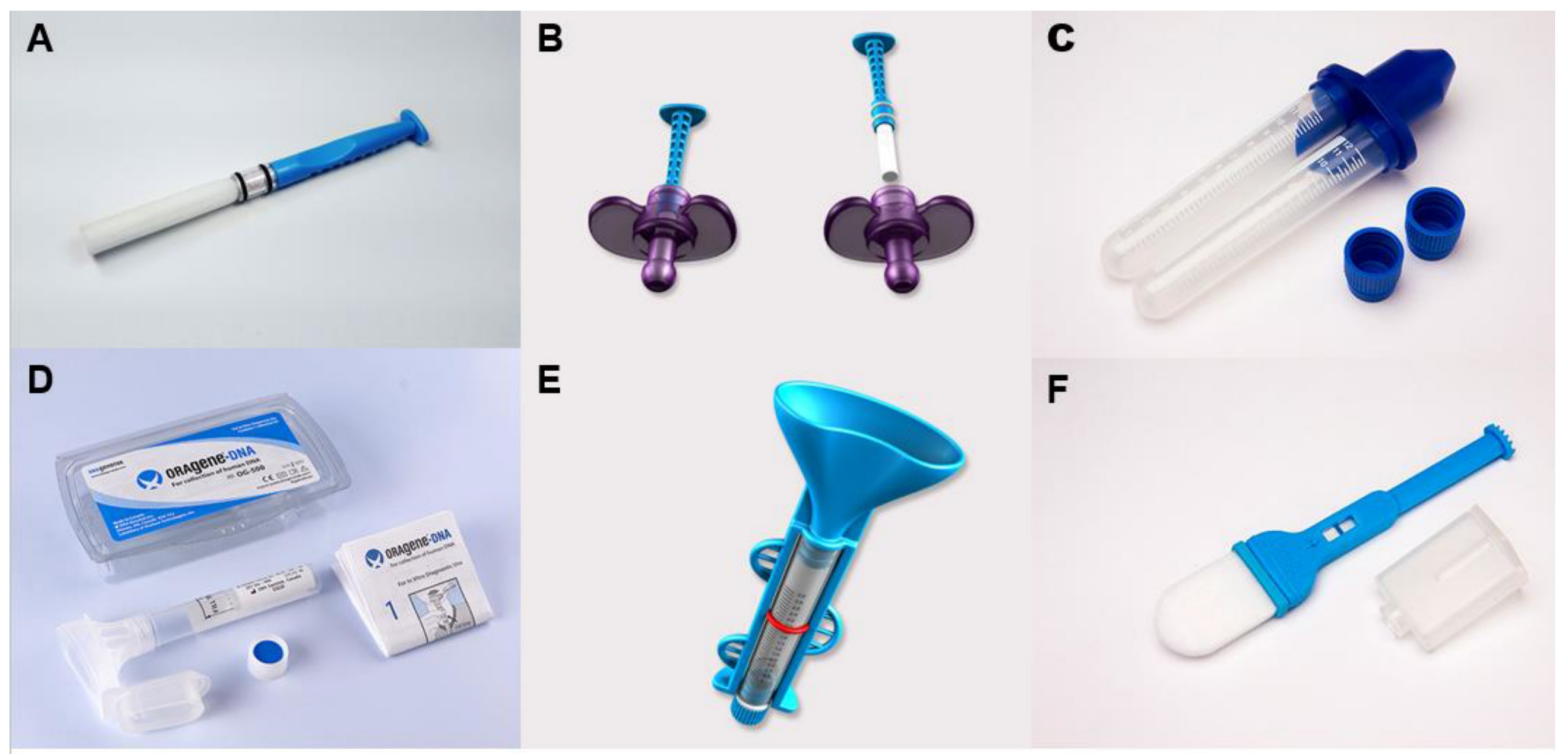
** Visual illustration of the deviations among commercially available salivary collection devices.** The devices depicted are as follows: A: Super•SAL™ Universal Saliva Collection Kit, B: Pedia•SAL™ Infant Salivary Collection, C: UltraSal-2™, D: Oragene DNA | OG-500, E: SimplOFy™, F: Versi•SAL® Saliva Collection Kit.

Methods for saliva collection have significantly advanced over the past decade, leading to standardized, reliable devices and techniques that collect high yield saliva within minimal variation. Commercialization of saliva collection kits was accelerated recently, due to the global pandemic of SARS-Cov-2, several companies obtained Emergency Use Authorization from the FDA for saliva collection and testing. Before saliva can be widely accepted as the medium of choice, collection (table 1) and protocols need to be robust. Due to the variation in consistency of saliva among collection sites and under specific physiological states, there are devices that can be utilised to maintain reliable saliva specimen collection. To fulfill this need, several products are commercially available, predominantly designated for research use (OraSure® Oral Fluid Collector, USA) and some of them have gained FDA approval (SDNA-1000 saliva collection device). As previously described, and illustrated in Figure 2, the site of origin of saliva production determines the contents. Common, commercially available saliva collection devices are illustrated and described in figure 3 and table 1, respectively. The specific variations of biomolecular contents and viscosity are vital to be kept consistent for the development of microfluidic devices. By ensuring the collection of saliva originates from desired site, aided with the collection devices available (as listed in table 1), the specifications of microfluidic devices can be optimized for desired biomolecule targets.

## Laboratory assays using saliva as a matrix

Saliva offers potential as an alternative diagnostic medium to blood based tests, due to the oral cavity's dense vascularization, circulating molecules are transported into saliva that can be utilized to detect underlying disease. Laboratory-based tests can quantify biomarkers of clinical relevance in saliva samples for a large number of oral and systemic diseases 35, 36, 47, 67-69. Biomarker detection is gaining attention in the medical field for monitoring physical conditions of the body. In general, monitoring physical condition-based biomarkers can be complex because of time dependent dynamics. Such biomarkers provide a snapshot of health status, and these are found in body fluids such as blood, saliva, urine and cerebrospinal fluid (CSF) 70, 71. Moreover, studies have demonstrated that changes in miRNA, DNA methylation, fitness parameters, and biomarker abundance can be quantified in saliva samples to assess underlying pathophysiological changes 21, 22, 72. More recently, with the current COVID-19 pandemic, saliva samples have been used to detect SARS-CoV2 and antibodies 2. With the rapid development in medical innovation devices, salivary testing for diseases has become more feasible in a laboratory setting and using commercially available self-use devices. Salivary diagnostic research overcame the precipice in 2013 when the Federal Drug Administration (FDA) approved the first self-test saliva antibody platform for human immunodeficiency virus (HIV)-1 and HIV-2 detection 45, 73, 74. More recently, CancerDetect™ from Viome received FDA approval under the breakthrough device designation to early predict the development of oral cancer and throat cancer 75. Further studies have utilised saliva samples to quantify hormone levels in individuals to assess health and disease status, brain function and development 44, 69, 76. Given the demonstrated diagnostic utility of saliva, its application has been further specialised into novel diagnostic methods for head and neck cancers of varying etiology 29, 30. Studies have further demonstrated that the salivary proteome can be quantified to diagnose and stratify HF patients 23, 36.

The current COVID-19 pandemic has lifted the salivary diagnostic field to new heights, with a clinical need for rapid POC diagnostic devices to efficiently diagnose patients with the rampant virus. To fulfill the need for rapid SARS-CoV-2 testing, there were numerous lab-on-chip (LOC) modalities specialised for virus testing. Reverse transcription loop-mediated isothermal amplification (RT-LAMP) is a nucleic acid amplification procedure that has been frequently used to detect bacteria and viruses 22, 77. A similar technology can be translated onto a paper-based analytical device is RT-LAMP, which produces a reverse transcription cDNA from an RNA model sequence, that is then replicated by DNA-polymerase 22. This technology does not require the laboratory facilities of traditional PCR amplification and can be carried out under the condition of 65℃. Adaptions of this technology is commercially available for the rapid detection of SARS-CoV-2, through a point-of-care colorimetric COVID RNA genome detection test 78, 79. Furthermore, SkiCell and Sys2Diag/CNRS have developed the EasyCOV SARS-CoV-2 platform to detect the virus using a RT-LAMP colorimetric experiment conveniently enclosed within a test tube 80. RT-LAMP POC platforms are widely accessible diagnostic tool, that has demonstrated a 70.9% sensitivity in detecting SARS-CoV-2 in patients, while the traditional RT-PCR remains the gold standard viral gene detection method with 81.6% selectivity 81. Thus, in the standard hospital setting the RT-PCR amplification method should be utilised, the POC RT-LAMP devices meet a clinical need for rapid, self-testing, without medical facilities at a low price point while utilising saliva as the diagnostic body fluid.

Antibody specific testing has been utilised in blood samples for decades and has remained as the gold-standard for biomarker validation analysis. Upon infection, antibodies are produced as an immunological response is mounted to combat - pathogens, these specified antibodies (immunoglobulin, Ig) can be detected and quantified using enzyme-linked immunosorbent assays (ELISAs). An ELISA is a highly sensitive and specific method to quantify the abundance of a target protein by capturing it in the ELISA complex that can be quantified through electrochemical or colorimetric output for result interpretation 82. Some studies suggest that IgG, IgA and IgM antibody quantity in saliva and serum are independently abundant in response to SARS-CoV-2, there is significant correlation to suggest the clinical utlity of saliva, with slight differentiation with Ig expression between time points of the body fluids 83, 84. An investigation into the variation of immunoglobulin variability revealed that IgM and IgG were reduced in abundance in saliva, IgA remained in high abundance and was detectable within 2-days of the onset of symptoms 85. The viability of salivary antibody analysis in an ELISA based POC device was demonstrated by the Brevitest IgA Salivary Mucosal Test (BRAVO) platform, which in a study upheld a negative and positive predictive score of 92% and 97%, respectively 22, 83.

## Challenges of using saliva in POC

Salivary diagnostics needs appropriate identification and validation of biomarkers that are specific for the underlying diseases. A biomarker is a quantifiable factor that can interact physiologically and biochemically at a molecular or cellular level, and can act as a surrogate indicator of normal, pathological, and interventional behaviors of the body's response. Furthermore, biomarker development and validation are emerging as an important component in translational studies, especially when developing diagnostic assays. Biomarkers can be used to early diagnose diseases, as well as to monitor response to treatment 86. Such biomarkers capture the health status of an individual and can be found in saliva samples. We discovered the world-first, 2 mm occult human papillomavirus (HPV) driven oropharyngeal cancer in an asymptomatic individual using salivary testing for HPV-16 as a biomarker 87, 88.

One of the major challenges using saliva as a matrix in a POC is the low abundance of analytes when compared to serum and plasma concentrations 89, 90. Historically, there were no sensitive detection platforms to detect analytes at pg/mL ranges in biological fluids (e.g., saliva) 50. This becomes a major issue when analysing analytes in saliva collected from children, where the concentration levels are very low 91, 92. However, modern technologies (digital PCR, highly sensitive immunoassays) are now able to quantify biomolecules in sub- pg/mL ranges 93, 94. These new technological advances enable saliva to be used as a matrix in a POC setting. In addition, the type of saliva, time of saliva collection, life-style factors, ethnicity, age, sex and other confounding factors can influence analyte levels 34, 95. As an example, when collecting saliva from patients who are dehydrated, saliva collection becomes difficult due to the mucous nature of saliva which can then be strenuous to pipet for down-stream applications 96. Furthermore, the mechanism of action of microfluidic biosensors involves capillary action that is governed by the fluid dynamics of a patient's salivary viscoelastic properties 97, 98. It is also equally important to standardize saliva collection methods to generate reproducible results for the quantification of analytes 50.

## Microfluidic devices for salivary analysis

The first part of this review focused on exploring the capacity of saliva as a diagnostic body fluid. Now the focus will be on POC microfluidics and the integration of saliva sampling into different types of POC microfluidic systems with various detection modalities.

In the field of Life Sciences, microfluidic devices are integrated instruments designed to quantify specific biomarkers in small volume physiological samples. Generally, microfluidic devices consist of microchannels that direct fluid flow creating a simple micro-sized operating system. These devices aim to perform traditional lab-based processes on a smaller scale. The miniaturization of these processes offers advantages over macro-scale systems, and these include consuming less reagents, shortening analysis time, and automating the diagnostic process. The automation of the process of analysis reduces operator error and creates a consistent, repeatable assay. Currently, microfluidic devices vary greatly in materials and detection methods, but this has not always been the case. In 1957, one of the first published microfluidic devices designed for biological diagnostics used an enzymatic-based test to measure glucose in urine. About 20 years later, the home pregnancy test became commercially available and is now one of the most well-known immunological microfluidic devices. However, both microfluidic devices were paper-based and produced a colorimetric readout. It was not until the seminal development of Stephen Terry's miniaturized gas chromatograph on a silicon wafer that the microfluidics' field got the first “lab-on-a-chip”. The advancement of microfluidic devices stem from miniaturization and microfabrication technology. For example, Andreas Manz in 1990 integrated microchip technology with the microfluidic system to introduce the concept of total chemical analysis system (µTAS). Decades later, new microfabrication techniques and biocompatible materials are still being created for novel applications of microfluidic devices.

Microfluidic technologies have been used in fields like lab testing, cell analysis, and environmental monitoring research, but for this review, we will focus on the application of microfluidics in POC medical diagnostics 99-105. POC testing allows medical analysis to be carried out near the site of patient care. Traditionally, medical testing requires sample collection and transport off site to a dedicated laboratory for analysis 106. The goal of POC testing (POCT) is to improve the quality of care for patients by providing relevant and useful results near the patient in a fast, timely fashion. Microfluidic technology offers a small size operation, portability, and shorter detection times making it essential for the development of POC testing technology. Also, microfluidic systems have high sensitivity and can obtain results rapidly which is important in POC testing. Successful development of microfluidic POC diagnostic devices would improve medical care in undeveloped and developed countries. Doctor's offices, patient clinics, and remote clinics frequently do not have the trained personnel or equipment to run needed diagnostic tests. Trained personnel are also not always available to collect invasive samples such as blood; therefore, non-invasive biological fluids, such as saliva, would improve on the ability of POCT to help affected individuals. Microfluidic systems have been developed to quantify salivary biomarkers with the goal of improving disease treatment in developed and developing worlds alike.

Many factors are contributing to the growth of the POCT market, such as technological advances and rising prevalence of communicable and noncommunicable diseases around the world. In particular, the Covid-19 pandemic has exposed the need for mass rapid POCT. The gold standard for Covid-19 diagnostic testing is reverse transcription-polymerase chain reaction (RT-PCR). While RT-PCR is a highly accurate method, it requires trained staff with equipped laboratories, and this diagnostic method usually takes at least 4 hours to complete. Newley developed saliva based microfluidic POCT devices, such as Abbott ID Now, are commercially available and can deliver results in 15 minutes or less.

Saliva-based microfluidic systems have been developed for detecting biomarkers related to cancer, cardiovascular disease, and more. As technology improves and researchers continue to optimize their microfluidic disease diagnostic platforms, the next generation of POCT regimes could improve healthcare delivery around the world. The developed microfluidic systems vary greatly in approach and design, especially in recognition elements and detection methods. In this review, we will focus on three types of microfluidic diagnostic platforms, specifically for saliva-based diagnostics: lateral flow assays (LFAs), LOC, and microfluidic paper-based analytical devices (µPADS).

## Lateral Flow Assays

LFAs, also known as “test strips,” are one of most widely used platforms for microfluidic diagnostics because they can be used to detect biomarkers in liquid samples without the need for specialized equipment or trained personnel. LFAs are typically comprised of sample inlet, conjugate, detection, and absorption pads, and each provide functions including sample loading, reagent storage, and analyte detection. Commonly, nitrocellulose and/or glass fiber are used for the pads, but the substrate can also be other woven materials. Sample transport through the porous pads via capillary action produces a qualitative colorimetric or fluorescent signal for read out. LFAs can be divided into two main categories - (a) lateral flow immunoassays (LFIA) and (b) lateral flow nucleic acid assays. The ability for LFAs to detect nucleic acids, antigens, and antibodies creates great potential when combined with saliva-based analytes 107-110. One of the most known and commercially available LFAs is the home pregnancy test. Commercial home-based pregnancy detection kits aim to detect the hormone, human chorionic gonadotropin (hCG), through a urine-based immunoassay. Recently, salivary hCG has been presented as a novel biomarker for early detection of pregnancy. In this study, saliva samples were tested on home-based pregnancy detection kits meant for urinary hCG, and results were confirmed and correlated with laboratory-based urine hCG and/or ultrasound examination. The study showed 77% accuracy and 23% false-negative results. The results using LFA designed for urine indicate that salivary hCG has the potential to become a biomarker for pregnancy detection. The company Salignostics claims their Salistick^TM^ is the first rapid saliva-based home pregnancy test for early and accurate pregnancy detection. This saliva-based test demonstrates a decrease in accuracy compared to traditional urine-based test, but the saliva diagnostic technology is relatively new 111. As technology and research advances over time, a saliva-based pregnancy test could compete with the accuracy a urine-based test and can offer advantages such as ease of sample without requiring a toilet.

Cortisol, the “stress hormone,” is a salivary biomarker of physiological stress, and this steroid also regulates varying physiological processes (e.g., metabolism, blood pressure, immune response) 112, 113. Irregular cortisol levels may also indicate an adrenal gland disorder. Adrenal gland disorders, including Cushing's Syndrome and Addison's Disease, if not treated, can lead to serious health issues. In general, cortisol LFAs are measured using competitive or sandwich immunoassays using a LFIA. Nardo et al. created a novel LFIA for salivary cortisol through direct and mediated coupling of antibodies to gold nanoparticles (GNP-Ab). Colloidal gold is the most commonly used label for commercially available LFIAs due to its high signal, long-term stability, and ability to be directly visualized. The covalent coupling approach produced superior results over adsorption techniques, but both sensors produced limit of detections (LOD) and range of detection required for clinical applications. The comparison of direct attachment of antibody probe and attachment of the antibody probe through protein A to the gold nanoparticle for the same assay offers valuable information. It shows the advantages and disadvantages of each approach. Moreover, Dalirirad et al. developed an LFA for detection of salivary cortisol using an aptamer instead of antibodies. The colorimetric LFA device used a duplex aptamer conjugated to Au nanoparticles (AuNPs). The first aptamer was a capture probe and the other used for binding of cortisol. The addition of cortisol containing samples created a conformational change causing dissociation of the aptamer from the capture probe. Increased dissociation leads to increasing binding of AuNP on test line; therefore, the test line color intensity increases as cortisol levels increase. Salivary cortisol was detected in the clinically relevant range of 0.5 - 15 ng/mL, and results were confirmed by enzyme-linked immunoassay (ELISA). Aptamers offer important advantages over antibodies. In general, aptamers have higher affinity to antigens and a longer shelf life than antibodies: therefore, aptamer-based sensors could be the focus of the next generation of POC diagnostics.

Extracellular vehicles (EVs) are lipid membrane structures secreted by all eukaryotic and prokaryotic cells. EVs play a key role in cell communication, transport of materials among cells, and regulation of cellular physiology. Dong et al. recently fabricated a nanosphere-based LFA capable of quantifying EVs in Saliva 92. The strategy combined membrane biotinylation using biotin-functionalized phosphatidylethanolamine (DSPE-PED-Biotin) and LFAs using fluorescent nanospheres as reporters for EVs quantification. The run time was under 1 hour and allowed for detection of 2.0x10^3^ particles/mL. The quantification performance of the assay was studied in saliva samples and produced a linear range between 4.0x10^3^ and 2.0x10^5^ particles/mL, indicating the saliva matrix had no substantial effect on the LFA.

LFAs have also been successfully developed for detecting several Schedule II drugs in synthetic and real saliva samples (e.g., cocaine, morphine, and methamphetamine) 114, 115. A cocaine LFA used a noncompetitive sandwich format composed of biomimetic material combined with gold nanoparticles conjugates, and the signal intensities correlated to cocaine concentrations were quantified using a smartphone 114. In 2018, Hu et al. developed an up-converting phosphor technology-based lateral flow assay (UPT-LFA) for POC testing detection of morphine and methamphetamine without sample pre-treatment. The UPT-LFA quantitative test out preformed liquid chromatography tandem mass spectrometry (LC-MS) with a lowest threshold efficacy of 2.0ng/dL 116. The UPT-LFA test for 50 simulated saliva samples was not only faster but had a higher detection efficiency than the LC-MS test. The UPT-LFA had a lowest threshold detection of (2.0ng/dL) with inter and intra-day analytical precision of <10%. The ability for a LFA to outperform a lab-based instrument decreases the overall diagnostic cost for those Schedule II drugs. Advantages and disadvantages of saliva, as a sample fluid, have been mentioned earlier in this review, but a specific disadvantage of saliva in a LFA and microfluidic paper-based analytical devices (µPADs) is viscosity. These microfluidic systems utilize the self-driving flow of capillary action to transport the sample throughout the device. Capillary flow is governed by the Lucas-Washburn equation:
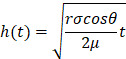
where µ is dynamic viscosity. Therefore, the viscosity of saliva sample changes the fluid dynamics of the entire system. Also, most LFAs produce a positive or negative signal that is interpreted by human eyes, allowing for readout error. The lack of quantitative accuracy for naked eye readout can be solved using an optical reader 117. Currently, the availability of smartphones does limit the possible outreach of POCT, but smartphones continue to become less expensive and more readily available. The integration of smartphones and POCT allows for more quantitative diagnostics and limit the need for visits to health care providers therefore decreasing cost.

## Lab on a Chip

The most technologically advanced microfluidic devices for POC saliva testing are Lab-on-a-Chip (LOC) devices. These devices make use of microfabrication to produce fluidic circuits (“chips”) able to preform laboratory functions for analyzing liquid samples as little as a few picoliters in volume. Through the integration of valves, pumps, sample reservoirs, electronics, and other components, LOC devices can preform sample pre-treatment, manipulation , and biomarker detection, essentially creating a miniature laboratory in a few square centimeters. The first LOC analysis system, a gas chromatography analyzer, was reported in 1979 by Terry et al. In the early years of LOC research and development, LOC systems were fabricated in silicon using photolithography, but fabrication materials have expanded to polydimethylsiloxane (PDMS), thermopolymers (PMMA, PS), glass, and paper that do not require as extensive facilities. LOC systems also allow for a variety of detection methods (electrochemical, colorimeteric, fluorescence, etc.) which, when combined with the different available device materials and fabrication methods, creates an attractive platform for developing POC biosensors. Currently, LOC biosensors have been successfully created for the detection and quantification of proteins, antibodies, nucleic acids, and organic molecules in saliva samples.

Polymers, due to their flexability and low cost, are the most popular material for LOC devices. Elastomeric PDMS is the most common polymer used because of the ability for rapid prototyping, gas permeability, optically transparency, and chemical inertness 118. Cheng et al created a PDMS-based LOC sensor for the rapid detection and quantification of cotinine, a biomarker used to determine the exposure to tobacco smoke, in saliva 119. Their microfluidic immunoassay only used 12 ml of sample and produced results in 40 min. The sensor had a linear detection range of 1-250 ng/mL. The correlation coefficient of the calibration curve (>0.99) confirmed correlation against laboratory-based tests. PDMS-based LOC systems have also been developed for glucose and cortisol, common salivary biomarkers 120, 121. The use of PDMS as the backbone of a POC microflidic system does offer disadvantages. PDMS is inherently hydrophocic, and this promotes protein absorption to the surface. Other disadvanges include poor compatablity with organic solvents and the ability for water to evaporate through the polymer. These characteristics do not affect all systems or assys, and many researchers believe the advantages of PDMS out weigh the faults. Some PDMS characteristics are optimal for POC systems such as the ability to replicate structures at the nanolevel, low cost, and optical clairity allowing for real time monitoring of the system.

Human immunodeficiency virus (HIV) is a life threatning virus and, over time, HIV causes acquired immunodeficiency syndrome (AIDS). HIV is easily transmittable; therefore, early detection and monitoring is crucial to limit or prevent spread. Recently, a multiplexed LOC system was developed by Chen et al. for the simultaneous detection of viral RNA and anti-HIV antibodies in blood and saliva samples. The microfluidic chemical and reagent device (CARD) is an autonomous system once sample and reagents are loaded. The disposable CARD utilises valves, pumps and resivories connected through microchannels to transport sample throughout the system in order to perform the complete assay. For the viral RNA detection, magnetic bead-based purification was proceeded by reverse transcriptase loop-mediated isothermal amplificaiton (RT-LAMP) assay to amplify the viral RNA. For the detection of anti-HIV antibodies, a lateral flow assay immunoassay (LFIA) with HIV glycoproteins is preformed. The fluorescence detection of both biomarkers required a run time around 80 minutes. This finalised system does require trained user intervention to load samples and reagents and specialized software controlled instrumentation, attributes not ideal for POCT. However, the abilitly to simultaneously detect antibodies and confirm a seropositive HIV-RNA reusult in a saliva sample validates the CARD as an important diagnostic system.

The versatility of LOC systems has allowed for novel sensors to be developed for several global outbreaks. For example, the Zika virus, spread by certain mosquitoes, currently has no vaccine; therefore, early detection allows for improved patient care 122. While the symptoms are usually mild and non-life-threatening, Zika can be passed from a pregnant woman to child and cause birth defects. In 2016, Song et al. created an “Instrument-free” POC microfluidic cassette for the molecular detection of Zika virus 123. The term “instrument-free” was used to denote without complex instrumentation. Their disposable cassette contained multiple independent amplification reactors, and each inlet was equipped with a silica-based nucleic acid isolation membrane. Nucleic acids are captured by the membrane and serve as templates in an RT-LAMP amplification process. The cassette is then incubated with a phase change material (PCM) to regulate temperature. The device was tested with raw saliva samples spiked with various concentrations and produced a sensitivity of 5 PFU of Zika virus per sample. The operation time was less than 40 minutes and produced comparable results without the need for a lab, electricity, or trained personnel. A simple straight channel device that does not depend on flow to sequentially run the assay offers a simple, repeatable POC device for saliva samples.

According to the CDC, cancer is the second leading cause of death in the United States, but the early detection can improve treatment success. LOC systems have been developed for salivary biomarkers related to, but not limited to, prostate and oral cancers 92, 124, 125. Most detection methods for prostate-specific antigen (PSA), a commonly used biomarker for prostate cancer, use serum and whole blood as the sample. The novel LOC sensor proposed employing a hybrid nanocomposite of graphene nanoplatelets with diblock co-polymers and gold electrodes (GRP-PS_67_-b-PAA_27_-Au) for electrochemical quantification of PSA in saliva. The optimized LOC sensor gave a PSA detection ranging from 0.1 pg/mL to 100 ng/mL and a lower limit of detection of 40 fg/mL. The miniaturized electrical impedance analyzer included a 3-5 minute response time, faster than previously proposed serum-PSA electrochemical sensors, and recorded data in real time. Other advantages included improved sensitivity and does not require additional redox electrolyte for electron exchange. Similar to prostate cancer, most early detection testing methods for oral cancer use sera samples for related biomarker quantification. Anti-p53 autoantibodies concentrations in saliva can be used for the early detection of oral cancer. Recently, an autonomous LOC system combining a microfluidic chip with a magnetic immunoassay was developed for measurement of anti-p53 in saliva. The LOC developed for rapid screening successfully detected the relative concentration range of anti-p53 with a detection limit of 4 ng/mL. Dong et al. also created a novel LOC sensor for the immunodetection of salivary biomarkers related to cancer, arthritis, cardiovascular disease, and inflammatory disease. The novel optical biosensor comprised of polythiophene-C_70_ organic photodetectors provided absorbance-based detection of IL-8, IL-1β, and MMP-8 proteins in spiked saliva. Further, the quantification of IL-8 and IL-1β were tested with unspiked human saliva samples were validated by two commercial ELISAs.

## Microfluidic paper-based analytical devices

Since the emergence of µPADs around 2007, µPADs have developed into an analytical diagnosis tool that has been the subject of extensive research. µPADs are patterned sheets of paper (commonly filter paper) consisting of hydrophilic microchannels surrounded by hydrophobic barriers. Generally, a µL-sized sample is added to a sample inlet, and liquid transport allows sample to wick through the porous paper by means of capillary force and evaporation. Guided by the hydrophobic barriers, the sample reaches a desired location/locations to produce a diagnostic readout. The cheap, disposable, and equipment-free properties of µPADs offer an ideal medium for mass producible point-of-care microfluidic devices. A majority of µPADS fabricated for bioanalysis are colorimetric assays based on enzymatic reactions or small molecule dyes.

Glucose, a simple carbohydrate, is commonly used as a serum biomarker for diabetics. Recently, salivary glucose has been proposed as a biomarker for diabetes mellitus. Specifically, salivary glucose and amylase levels have exhibited a direct correlation to blood glucose levels in patients with diabetes mellitus. Researchers created a variety of µPADs for detecting salivary glucose 126. Santana-Jimenez et al. created a system fabricated from #40 Whatman^TM^ filter paper and a stamping procedure to implement the wax. The µPAD utilized the coupling of GOx-HRP with 2,4,6-tribromo-3-hydroxybenzoic acid for the detection of glucose in buffer and artificial saliva solutions. The proposed device, modified with chitosan to improve optical readout, produced a colorimetric signal suitable for naked eye detection. This bienzymatic sensor showed a LOD of 0.09 mM and a linear range of 1 to 22.5 mg/dL The research also reported analysis of human saliva samples without pre-processing resulting in recoveries from 92 to 114% therefore claiming potential for the device to be a fast, sensitive system for the detection of salivary glucose without device or trained personnel.

Nitrite and nitrate are biomarkers associated with cancer and other oral diseases. Traditionally, these ions are measured in blood or serum, but Ferreira et al. developed colorimetric µPADs for detecting salivary nitrite and nitrate using the Griess reaction 127. The nitrite µPAD consists of two paper discs sandwiched in a plastic laminating pouch. The top disc is unmodified for sample addition and the bottom or reagent discs contain the dried Griess reagent. Sample is added through a small hole in the laminating layer and flows from sample pad to reagent pad creating a pink color. After 4 hours, the device is scanned using a standard scanner and the resulting image is processed using ImageJ to determine the intensity of the shade of pink which is directly proportional to the nitrite concentration. The µPAD exhibits a nitrite determination range of 5 - 250 µM with a limit of detection of 0.05 µM and limit of quantification of 0.17 µM. The µPAD for nitrate determination contains three paper discs between a plastic laminating pouch. The top disc contains a zinc suspension; the other two discs are empty paper disc (middle) and a reagent paper disc (bottom). After 2 hours, the µPAD for nitrate determination is quantified through the same process producing a nitrate determination range of 0.2-1.2 mM with limit of detection of 0.08 mM and limit of quantification of 0.27 mM.

Thiocyanate is a saliva biomarker indicating for tobacco smoke exposure. In 2016, a µPAD was developed for detecting thiocyanate using the formation of an iron (III)-thiocyanate complex. The device used a previously reported fabrication process, and the signal was measured using a scanner and image processing software. Under optimal conditions using standard solutions made with ultrapure water, the µPAD produced a linear dynamic range between 0.25 and 20 mM of thiocyanate and a limit of detection of 0.06 mM with an R^2^ of 0.9995. The µPAD was tested with saliva samples and compared to a spectrophotometric reference method, and the compared results showed no statistically significant differences.

Due to the several orders of magnitude, a decrease in biomarker concentrations found in saliva versus blood, colorimetric µPADs are limited by detection sensitivity and for small molecules. This problem has been addressed by several in the field using a readout device such as a smartphone to aid in quantification of produced signal, but this solution is not practical for resource limited settings. Electrochemical detection is a commonly used analytical technique used in bioanalysis. Electrochemistry uses potential, charge, or current measurements to determine analyte concentration within a sample. Many µPADs sensors employing electrochemical detection methods have been proposed for a range of salivary analytes including glucose (carbohydrate), cholesterol (lipid), tetrahydrocannabinol (small organic molecule), and others 128-132. µPADs sensors have also made use of other detection methods such as fluorescence, but saliva is not typically the sample medium. Lee et al. recently developed an enzyme-loaded paper-based impedimetric sensor capable of detecting total cholesterol with a detection limit lower than the clinical levels in saliva. The sensor combined nitrocellulose paper with Pt black/Nafion composite to detect total cholesterol values in the range of 5-4000 ng/mL with a 0.99 linear correlation.

The continued legalization of medical and recreational cannabis use has created a need for a POC saliva-based roadside test capable of rapid and sensitive detection of Δ-9-tetrahydrocannabinol (THC). Renaud-Young et al. sensor allowed THC to infuse carbon paper electrodes before taking cyclic voltammetry measurements. The oxidation peak current varies with THC surface densities allowing for a linear dose dependent change able to detect THC concentrations below what is considered an impairing concentration. Moreover, a µPAD system using colorimetric and electrochemical impedance spectroscopy (EIS) was developed for the detection of influenza virus, the virus responsible for causing flu 133. The paper-based immunoassay was found to be sensitive and selective with a turnaround time of only 6 minutes. The device is constructed with altering layers of paper pads and adhesive tape to create a vertically flowing system operating similarly to an LFA. The use of a different pore size pad was used for the flow control of the sample and as a filter for the desired antigen. While this device does require a few user steps, the coupling of these two detection methods for the same analyte can reduce false results and provide more confidence in the POC diagnosis. The uniqueness of µPADs and saliva as a sample creates great potential for advancement of POC testing, but the combination of paper and saliva also presents obstacles. One limitation is a result of a previously mentioned advantage, the pump free nature of µPADs combined with the varying physical properties of saliva could impact the consistency of the dynamics of the system. The sample dynamics are governed by capillary action and evaporation: two processes directly related to the sample's viscosity. Other drawbacks include potential loss of analyte to the paper itself and low sensitivity of some detection methods.

## Summary and future directions

To minimise exposure to contagions such as seasonal influenza and SARS-CoV-2, we need testing options that can be performed in a field setting 134. POC detection technologies can be used in a field setting enabling decentralized, rapid, sensitive, low-cost diagnostics that are urgently needed to overcome global challenges associated with pandemics. POC detection technology would incorporate a biomarker that can be readily available in a biofluid, and the detection technology can either be optical or magnetic.

Salivary diagnostics is attracting increasing attention in the field of POC due to its non-invasive nature, reduces the discomfort and risk posed to the patient, can be collected without specialised training (or self-collected by the patient) and stored at room temperature for transportation, allowing greater accessibility for screening in rural, Indigenous, and regional communities 14. Saliva is also a more stable and a less complex matrix compared to blood and as such, is ideal for field testing 52. The launch of the world-first, biosensor to measure glucose levels in Type 2 diabetic patients has opened a new era of saliva-based POC diagnostics 135-137. Despite the progress in the field, significant challenges and opportunities still remain, particularly as more clinical studies are published linking biomarker levels in saliva to different disease states. One of the major challenges in using saliva in POC is due to its inherent viscoelastic properties. The major challenges that exist using saliva as a matrix largely depends on handling the complexity of saliva. The large range of molecular species combined with variation in viscosity and contents make saliva samples nearly as challenging as whole blood. Selecting key molecules and standardizing for natural variations in saliva represent key opportunities moving forward.

Microfluidic devices for saliva analysis have risen from a small, highly specialized application to one of more broad general interest over the last decade. Interest in saliva has grown specifically as more and more clinical studies have linked biomarker levels in saliva to those in blood. Likewise, more and more microfluidic devices have been developed specifically to handle saliva as a sample matrix. Personalised medicine will improve the primary healthcare system through the incorporation of biosensors, lab-on-chip systems, individual genetic consultation for therapy, smartphones monitoring parameters, and microfluidic devices. These transitions will enable clinicians to non-invasively monitor patient health, while remaining accurate, more consistent, capture clinical data quickly, provide patient satisfaction, and streamline workflows. Salivary diagnostics' impact on the healthcare system is potentially enormous, being more accessible, less cost intensive, non-invasive, convenient, and well-credentialed. The future development of this field with the further development of bioinformatics will improve testing standards and performance of tests. Moving forward in the field, several opportunities remain to both expand use and address key challenges for microfluidic-based saliva diagnostics. One of the major challenges with saliva is dealing with the complex and variable viscosity of the sample matrix. Viscosity can change by an order of magnitude between samples. At the same time, viscosity affects many aspects of the microfluidic assays ranging from flow rates to binding times. In addition, viscosity is chemically complex so addressing methods to maintain representative samples while still providing selective analysis is key. Finally, it is critical that continued development of validated biomarkers is essential. While several biomarkers have been validated, the number is far below that of blood-based biomarkers.

There is clear evidence of disparity in health care outcomes between individuals who live in urban centres and those in rural or Indigenous communities. Access to healthcare delivery services is hampered by the unavailability of diagnostics that can transcend the geographic and social barriers. As modern medicine is shifting towards prevention and early detection of several diseases, prior to the manifestation of morbid illnesses. The development of small wireless devices has and will continue to make a dramatic impact the healthcare services. The next decade will bring breakthroughs in terms of precision, efficiency, and bedside monitoring instead of hospital setups. In addition, the lack of screening and monitoring for diseases that are prevalent in these populations has led to reshaping healthcare delivery, using innovative diagnostic tools that use minimally invasive sampling. In addition, by using saliva, we allow disease surveillance to reach more individuals in a cost-effective manner using fewer human resources than blood collection procedures.

## Figures and Tables

**Figure 1:**
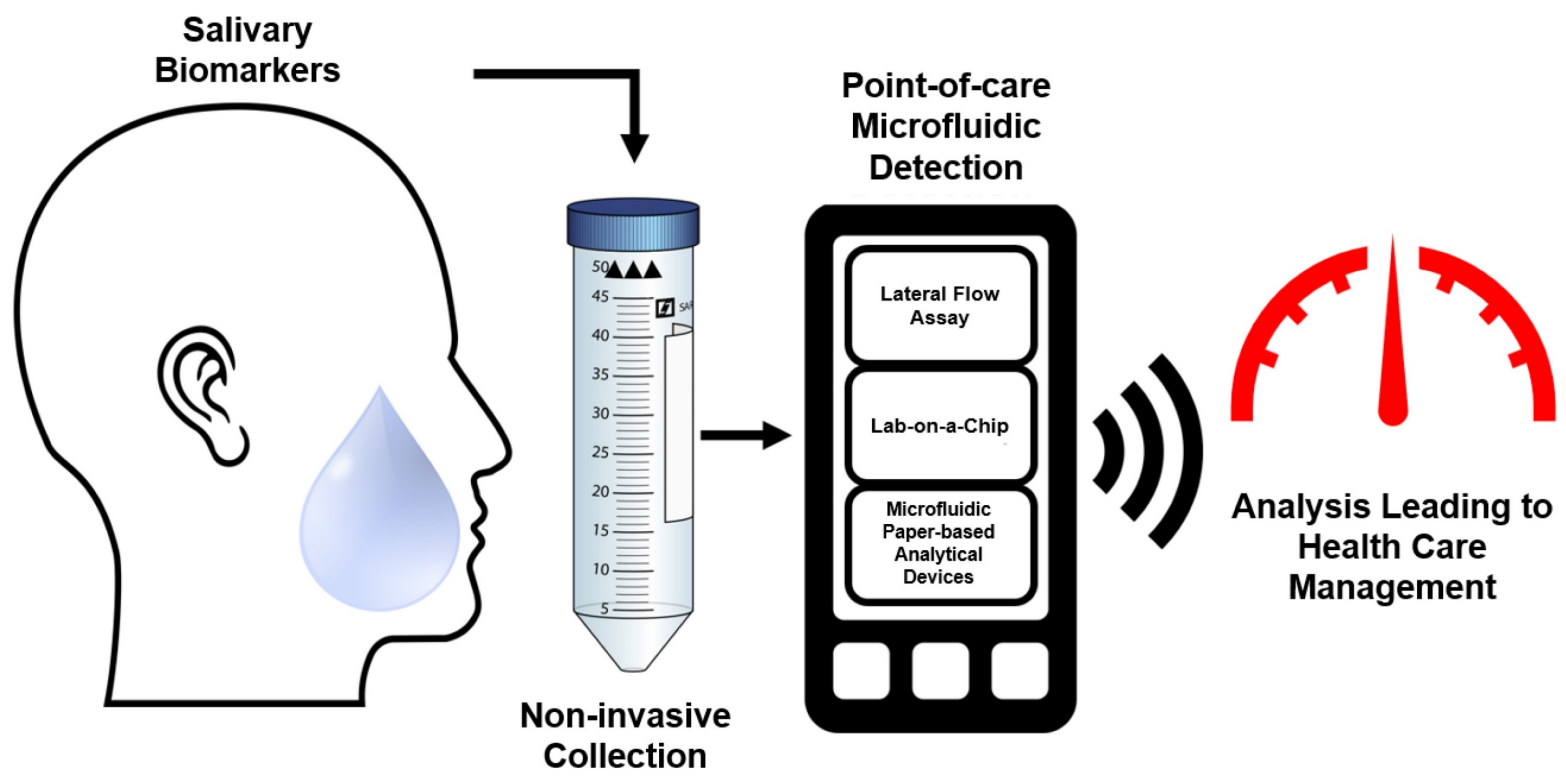
A visual abstract outlining the current unmet clinical need in the development of POC microfluidic devices when using saliva as the body fluid.

**Figure 4:**
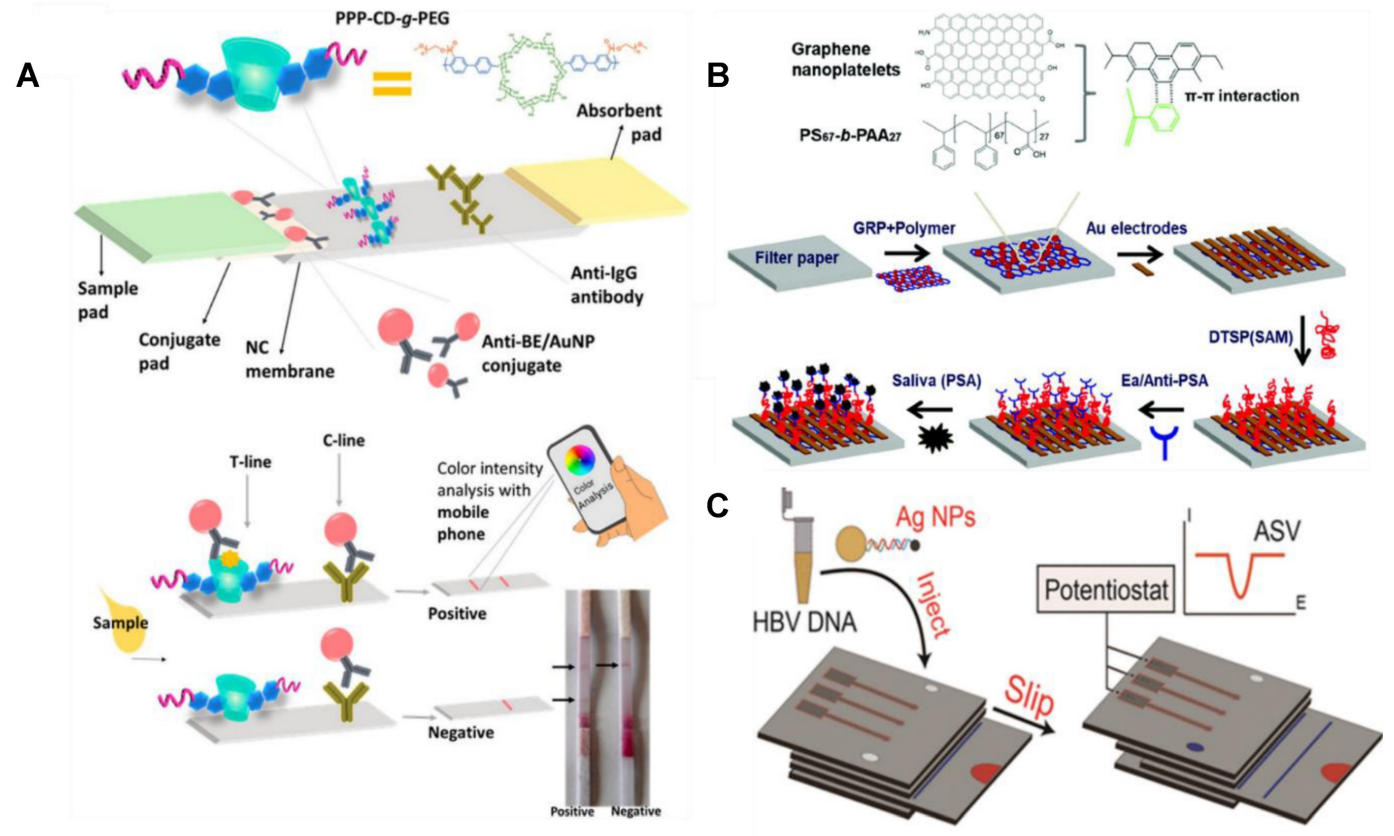
** Pictorial representations of three different microfluidic devices developed for salivary analyte detection. (A)** A LFIA for the detection of cocaine with the ability for color intensity analysis through a mobile phone. **(B)** A µPAD for electrochemical quantification of prostate specific antigen. **(C)** Another µPAD with the ability for electrochemical detection of Hepatitis B viral DNA detection. Adapted with permission from 114, 124, 130, copyright 2017,2018,2015.

**Table 1:** ** Table encompassing commercially available salivary collections devices.** The devices are listed by the manufacturing company, product name, type of saliva being targeted for collection and collection method.

Company	Device	Saliva Type	Collection method	Reference
ThermoFisher	SpeciMAX™ Saliva Collection Kit	Whole saliva	Drool	https://www.thermofisher.com/order/catalog/product/A50696P10
Spectrum Solutions™	SDNA Saliva Collection Device	Whole Saliva	Drool	https://spectrumsolution.com/sdna-whole-saliva-dna-collection-devices/
ZYMO Research	DNA/RNA Shield SafeCollect Saliva Collection Kit	Whole Saliva, stabilised in DNA/RNA Shield	Drool	https://www.zymoresearch.com/collections/saliva-collection
Salimetrics	SalivaBio Passive Drool Method	Whole Saliva	Drool	https://salimetrics.com/collection-method/passive-drool-saliva-collection-device/
Salimetrics	SalivaBio Oral Swab (SOS/SCS/SIS) Method	Mucinous and Serious Saliva	Swab	https://salimetrics.com/collection-method/oral-swab-saliva-collection-device/
DNA Genotek	OMNIgene®·ORAL (OME-505)	Whole Saliva, sample stabilisation for microbial nucleic acids	Drool	https://www.dnagenotek.com/us/products/collection-infectious-disease/covid-19-collection-kits/index.html
DNA Genotek	ORAcollect®·RNA (*ORE-100)	Mucinous and Serious Saliva, stabilisation for microbial nucleic acids	Swab	https://www.dnagenotek.com/us/products/collection-infectious-disease/covid-19-collection-kits/ORE-100.html
DNA Genotek	Oragene OG-500	Whole Saliva, stabilises DNA	Drool	https://www.dnagenotek.com/ROW/products/collection-human/oragene-dna/500-series/OG-500.html
ThermoFisher Scientific	SpeciMAX Saliva Collection Kits	Whole saliva, virus-inactivation and stabilisation	Drool	https://www.thermofisher.com/au/en/home/life-science/dna-rna-purification-analysis/sample-collection/specimax-saliva-collection-kit.html
Norgen Biotek	Saliva RNA Collection and Preservation Devices	Whole Saliva, anti-infectious and RNA preserving agent.	Drool	https://norgenbiotek.com/product/saliva-rna-collection-and-preservation-devices-dx-0
Norgen Biotek	Saliva DNA Collection and Preservation Devices Dx	Whole saliva, anti-infectious and RNA preserving agent.	Drool	https://norgenbiotek.com/product/saliva-dna-collection-and-preservation-devices-CE
NeuMoDx	100500 NeuMoDx™ Saliva Collection Kit	Whole saliva, specimen stabilisation (SSB)	Drool	https://www.fda.gov/media/145411/download
CD Genomics	MicroCollect™ Saliva Collection Devices	Mucinous, Serious, and Whole Saliva	Drool/Expectorate	https://www.cd-genomics.com/microbioseq/microcollect-saliva-collection-devices.html?gclid=Cj0KCQjw3IqSBhCoARIsAMBkTb2TLKjRq9K402aUuUONnRauLPw2LXSmogrAiY7qWe6drOK0LaljbpoaAhN-EALw_wcB
ASTRAL Scientific	GeneFiX Xtra Saliva DNA & RNA Collectors	Whole Saliva, Stabilises DNA and RNA	Drool	https://astralscientific.com.au/collections/saliva-dna-collection-and-isolation/products/genefix-xtra-dna-saliva-collectors-3ml
SUPER•SAL™	Universal Saliva Collector (with Compression Tube	Whole saliva	Collection Pad	https://www.filgen.jp/Product/Bioscience4/Oasis/SSAL-601.pdf
PreAnalytiX	PAXgene ® Saliva Collector	Whole saliva	Drool	https://www.preanalytix.com/products/saliva/dna/paxgene-saliva-collector-mba/US?cHash=2319179f194effee506bf153b79554d2&cmpid=CM_QF_SPD_PAXgeneSalivaCollector_1021_SEA_GA&gclid=Cj0KCQjw_4-SBhCgARIsAAlegrWZ_uBoPHN80EvcrBblqeYO7FczUhcbAnW7LY-tJfdut367gh15XQAaAvixEALw_wcB
CROCOMed	Single-use samplers (Individual package/Inactivated 10ML)	Whole saliva with antigen inactivation	Drool	https://www.crocomed.com/ProductList/info.aspx?itemid=92
Suntrine	Viral transport medium tube (saliva samplecollector)	Whole saliva, with preservation solution	Drool	https://en.suntrine.com/product/viral-transport-medium-tube-with-swab-saliva-sample-collection/
RXBio	SDNA-2000/3000	Whole saliva, with viral RNA stabilisation	Drool	https://rxbio.co/ppe/spectrum-saliva-kit/
Qiagen	PAXgene Saliva Collectors	Whole Saliva	Drool	https://www.qiagen.com/us/products/discovery-and-translational-research/sample-collection-stabilization/dna/paxgene-saliva-collector/
PanoHealth	Saliva Collection Kit	Whole saliva	Drool (oral rinse)	https://panohealth.com/wellness-services/saliva-collection-kit/
Canvax	Saliva Sample Collection & Stabilization Kit	Whole Saliva	Drool	https://lifescience.canvaxbiotech.com/product/saliva-sample-collection-kit/
Cambridge Bioscience	Saliva collection kit with DNA/RNA Shield	Whole saliva, with DNA/RNA stabilisation and preservation	Drool	https://www.bioscience.co.uk/cpl/saliva-collection-kit
Biolinkk	DNA/RNA SHIELD SALIVA SPUTUM COLLECTION KIT - DX	Whole Saliva, with DNA/RNA preservation (nucleic acids)	Drool	https://biolinkk.com/product/dna-rna-shield-saliva-sputum-collection-kit-dx/

**Table 2:** ** List of Saliva-based microfluidic devices reported in the literature.** Also given is assay type, specific biomarker, and associated illness.

Platform	Assay Type	Analyte	Associated Illness	Source
LFA	Colorimetric	Cortisol	Adrenal gland disorders	138
LFA	Colorimetric	Cortisol	Adrenal gland disorders	139
LFA	Fluorescence	Extracellular vesicles		140
LFA	Colorimetric	Cocaine	Substance Dependence	114
LFA	Colorimetric	Morphine and methamphetamine	Substance Dependence	115
LOC	Fluorescence	Cotinine	Tobacco smoke exposure	119
LOC	CARD	Viral RNA and anti-HIV antibodies	HIV	141
LOC	Fluorescence	Viral RNA	ZIKA	142
LOC	Colorimetric	ZIKA virus	ZIKA	123, 143
LOC	Electrochemical	Prostate-Specific Antigen	Prostate Cancer	124
LOC	Colorimetric	Anti-p53 autoantibodies	Oral Cancer	125
LOC	Absorbance	IL-8, IL-1β (proteins)	Cancer, arthritis, and cardiovascular disease	92
µPAD	Colorimetric	Glucose	Diabetes	126
µPAD	Electrochemical	Glucose	Diabetes	128
µPAD	Colorimetric	Nitrite and nitrate	Oral diseases	127
µPAD	Colorimetric	Thiocyanate	Tobacco smoke exposure	144
µPAD	Electrochemical	Cholesterol	Heart disease	129
µPAD	Electrochemical	Tetrahydrocannabinol (THC)	Substance Dependence	131
µPAD	Colorimetric and electrochemical	Influenza virus	Flu	133
